# Identifying Gaps in the Understanding of Eating Disorders Amongst Medical Students Across South Wales Using a Cross Sectional Survey

**DOI:** 10.1192/bjo.2025.10473

**Published:** 2025-06-20

**Authors:** Rhiannon Kihara, Thomas Prew, Umer Jalal, Lois Bown, Isabella Jurewicz

**Affiliations:** 1 Swansea Bay University Health Board, Swansea, United Kingdom; 2 Cardiff and Vale University Health Board, Cardiff, United Kingdom; 3 Cardiff Medical School, Cardiff, United Kingdom

## Abstract

**Aims:** Eating disorders are complex, serious illnesses that can result in physical and psychiatric co-morbidities, medical emergencies and progressive health consequences. The aim of this service evaluation was to explore current knowledge and understanding of eating disorders amongst medical students in South Wales, and evaluate current teaching and training.

**Methods:** Two separate cross-sectional web surveys were designed for final year medical students at Swansea and Cardiff Universities using Microsoft Forms. Participation was voluntary, and anonymised. Surveys consisted of eight Likert-based questions and one free text question, allowing participants to share personal details should they wish to participate in future data collection. The survey was disseminated via email between 7 and 14 October 2024.

**Results:** A total 16 final year medical students from Swansea and 21 from Cardiff completed the surveys.

Over 80% of medical students reported low confidence (rated as 5 or below /10) in their ability to describe the seven types of eating disorder. 90% of students from Cardiff and 75% of students from Swansea reported low confidence in their knowledge of the prevalence and their ability to describe a medical risk profile. 62% from Cardiff and 44% from Swansea reported low confidence in their ability to elicit symptoms of eating disorders and make diagnoses. As a result, only 38% of Cardiff students and 56% of Swansea students reported feeling confident (rated 6–10 /10) to assess the needs of patients with eating disorders and communicate with them effectively. Over 75% of the entire student cohort described low confidence in their ability to identify stages and types of management for eating disorders. Fewer than 10% of students from both Universities felt highly confident (8–10/10) that they would be able to describe medical emergencies in eating disorders.

The entire student cohort from Cardiff and over 80% of students from Swansea expressed dissatisfaction with the education and training provided on eating disorders.

**Conclusion:** There are significant gaps in medical students’ understanding of eating disorders and confidence in assessing and managing eating disorders is low. Eating disorders may be stigmatised, and this may introduce additional barriers to teaching and to clinical exposure.

This service evaluation highlights the need for a review of medical school curricula to confirm the provision of eating disorder teaching.

More effective and comprehensive teaching, and clinical exposure will be indicated in order to improve confidence and competence in the assessment and management of eating disorders amongst medical students before graduating.

